# Survival at 30 days in elderly patients with hip fracture surgery who were exposed to hypothermia: Survival study

**DOI:** 10.1097/MD.0000000000027339

**Published:** 2021-10-01

**Authors:** Sergio Charles-Lozoya, Héctor Cobos-Aguilar, Edgar Manilla-Muñoz, Miguel Leonardo De La Parra-Márquez, Adrián García-Hernández, Jesús Mario Rangel-Valenzuela

**Affiliations:** a Health Science Division, Division of Plastic and Reconstructive Surgery, Unit of Hip and Pelvis Orthopedic Surgery, Hospital de Traumatología y Ortopedia No. 21, Instituto Mexicano del Seguro Social, Monterrey, N.L., México; b Health Science Division, Vice-rectory of Health Science, Universidad de Monterrey, San Pedro Garza García, N.L., México; c Graduate Baylor University, Private Practice, Dallas, Tx.

**Keywords:** elderly, hip fractures, hypothermia, mortality, survival

## Abstract

The effect of hypothermia as a mortality risk factor at 30 days in the elderly who had hip fracture (HF) surgery is still controversial because it may be due to a set of poorly identified factors. In this study, we aim to determine if exposure to intra and immediate postoperative hypothermia increases the incidence of mortality at 30 days in elderly patients who had HF surgery.

Survival study in the elderly who had HF surgery with and without exposure to hypothermia. Sociodemographic, anesthetic and surgical factors were collected. The temperature of the rectum was measured at the end of the surgery and in the recovery room. The effect of hypothermia was analyzed by the incidence of mortality at 30 days. Other results were considered, such as, surgical site infection (SSI), blood transfusions, and influence of implants used in the 30-day mortality.

Three hundred eighty five subjects were eligible, to include 300. Inadvertent hypothermia was 12%, the 30-day overall mortality was 9% and in subjects with hypothermia it was 25% (*P* = .002). Subjects with hypothermia had a higher risk of SSI (relative risk 4.2, 95% confidence interval 1.3–13.6, *P* = .03) and receive more transfusions (relative risk 3.6, 95% confidence interval 2.0–6.5, *P* < .001).

Elderly subjects with HF exposed to hypothermia who undergo hip hemiarthroplasty and who receive 2 or more blood transfusions during their treatment, are at greater risk of dying after 30 days of the surgery. Hypothermia, as a possible causative factor of mortality, should continue to be studied.

## 1 Introduction

Hip fractures (HF) are a public health problem due to its complications, which include chronic pain, disability, decreased quality of life and premature death.^[1]^ In 1990 the incidence of HF worldwide was 1.7 million and is projected to increase to 6.3 million in 2050.^[2]^ The mortality of HF matches that of pancreatic cancer, stomach cancer, and heart attack.^[3]^ HF has a one-year mortality rate of 37.1% and 26.4% for men and women, respectively.^[4]^ This high mortality may be due to cardiovascular, pulmonary, and infectious or neoplastic complications.^[5]^ Similarly, sex, body mass index, advanced age and smoking are factors associated with mortality at 3 and 12 months after the occurrence of HF.^[6]^ Patients may inadvertently develop hypothermia during and after surgical fixation of HF.^[7]^ Intraoperative hypothermia may decrease wound healing, increase the risk of surgical site infections (SSI) and increase intraoperative bleeding and cardiovascular morbidity.^[8]^ A reduction in core body temperature increases oxygen consumption and increases stress on cellular processes, resulting in high mortality.^[9]^ Therefore, blood loss, peripheral vasoconstriction and decreased cardiac output, cause imbalance between the demand and supply of oxygen, especially in vital organs; and in the elderly with cardiovascular disease, heart failure and acute myocardial infarction may occur during postoperative hypothermia.^[10,11]^ Despite heating protocols during the perioperative phases, there are several factors that cause hypothermia; such as, general anesthesia that inhibits the reflections of thermoregulation, the administration of intravenous fluids used to irrigate the wound, the temperature in the operating room; the nature, extent and duration of the surgical procedure, age over 60, low body weight, and American Society of Anesthesiologists (ASA) Classification.^[12–14]^ Hypothermia results in poorer outcomes, but its effect on HF patients is not well defined. The main objective of the study is to assess the morbidity and mortality associated in elderly patients who undergo HF surgery.

## 2 Methods

### 2.1 Patients

We performed a prospective survival study of consecutive HF surgeries during the period from December 2017 to December 2019 in the hip service of the High-specialty Medical Unit of the Trauma and Orthopedics Hospital No. 21 in Monterrey, Mexico.

After receiving approval from the local Research Committee (1903) and by the Ethics Committee (1903–8) from the Mexican Institute for Social Security (#R-2017–1903-11, date: 12-15-2017) and after written consent was obtained patients were included in the study. Inclusion criteria were male and female patients ≥60 years of age undergoing HF surgery, which was defined as a fracture between the articular cartilages of the hip up to 5 cm below the distal part of the lesser trochanter, diagnosed by orthopedics hip surgeons. Exclusion criteria were presence of multiple fractures, ASA^[15]^ Grade ≥IV, multiple organ failure or inability to provide informed consent. A sample size to compare 2 survival curves was use. $E=\frac{{\left(CTR+1\right)}^{2}(K)}{C{\left(TR-1\right)}^{2}}$; and to determine the number of subjects to study per group, it use, $n=\frac{E}{2-(\pi_{1}-\pi_{2})}$, with a ratio of unexposed to exposed of 6:1;^[16]^ frequency of mortality and exposure to hypothermia of 10.9%,^[12]^*δ*: .05 for a total minimum of 36 in the exposed group and 216 in the unexposed group.

### 2.2 Outcome definition

Rectal temperature was taking as the reference for the central temperature, because is a method with acceptable accuracy and is not as invasive as other temperature measurements.^[17]^ But like rectal temperature is lower, hypothermia was defined as temperature <35°C (95°F).^[18]^ Secondary outcomes included mortality and SSI at 30-day follow-up.

### 2.3 Temperature measurement and thermal care

It was performed by a nurse with a flexible digital thermometer (Omron MC-343F. Tokyo, Japan), which was delivered and calibrated by the supplier. It was performed 2 temperature measurements: immediately after surgery and upon arrival at recovery room, taking the average as the final measurement. Routine thermal care was delivered by the anesthesiologist discretion according to the following protocol: the thermostat in the operating room is set to approximately 21°C, intravenous fluids and blood were warmed, intraoperatively, the patient was covered above and below the field with 1 layer of paper surgical drapes. Postoperatively, either 1 or 2 cotton blankets were placed over the patient and a heated mattress was used.

### 2.4 Data collection

The following data was obtained from the patient and medical record: age, sex, ASA score, body mass index in agreement to Quetelet,^[19]^ diagnosis of diabetes mellitus or systemic arterial hypertension, central temperature, wetting time from the HF to surgical fixation, fracture type, and implant used: dynamic hip screw, proximal femoral nail (PFN), hip hemiarthroplasty (HHA), total hip replacement (THR) cemented and not cemented, plates, and dynamic condylar screw. From the anesthesia records the following data were extracted: bleeding in milliliters, average blood pressure, total surgery time, and number of transfusions. Records were made of the SSI that occurred 30 days after the surgery and were defined according to the infection prevention guide of the Centers for Disease Control and Prevention.^[20]^ Mortality was collected by institutional social workers, contacting caregivers and asked if the relative had died 30 days since the operation, or in they family medicine unitthis, this was explicitly stated in the study's consent form.

### 2.5 Statistical analysis

Mann–Whitney *U* test was used to contrast non-parametric variables; Pearson's χ^2^ test for comparison of frequencies and percentages of the dichotomous qualitative variables. To compare the risks, the relative risk was calculated (confidence interval .95). To measure the time for the occurrence of the post-hypothermia mortality event, the Kaplan–Meier and log-rank survival curve were used. To adjust the confounding variables, a Cox regression analysis was performed. *P* values <.05 was considered significant. All statistical analyzes were performed on SPSS (IBM SPSS Statistics, Version 25.0. Armonk, NY: IBM Corp.).

## 3 Results

Three hundred eighty five subjects underwent HF surgery during the study period. Forty two patients were classified as ASA IV, 18 as ASA V, 16 patients had other fractures and 9 had severe comorbidities, leaving 300 patients for further analysis. The mean age was 78.9 ± 8.7 years, with 210 females (70%). Of the total fractures, 132 were pertrochanteric (44%) and the most used implant was dynamic hip screw (n = 108, 36%).

**Figure 1:**
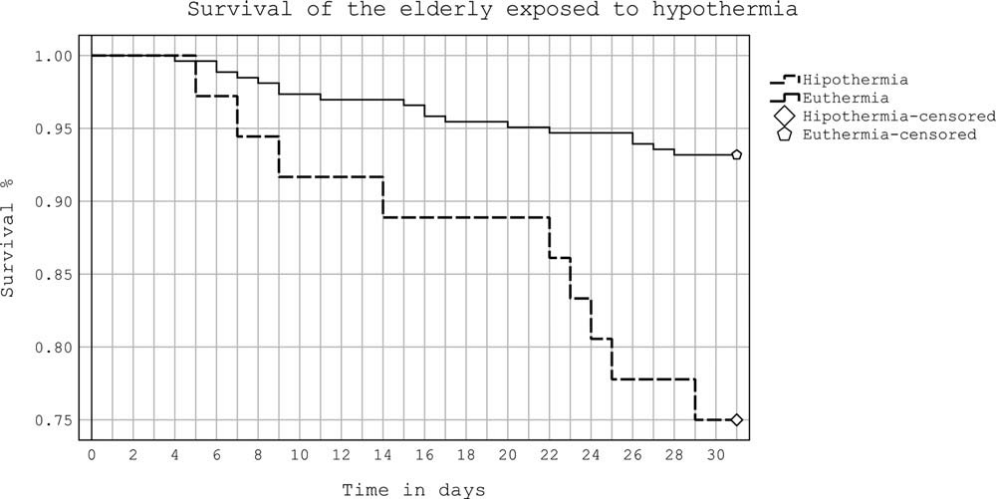
Kaplan–Meier survival curve, from 30-day mortality in two cohorts of subjects with and without inadvertent hypothermia, who underwent hip fracture surgery. The log-rank test was χ^2^ = 13.302, *P* < .001.

Patients who were hypothermic had higher mortality and SSI (*P* = .002), (*P* = .03) (Table 1). Patients who receive blood transfusions were more likely to be hypothermic (19 vs 28, *P* < .001). It was observed that the subjects classified as ASA III, presented a higher risk of hypothermia (*P* < .001) (Table 2). In an analysis between subjects who were or were not transfused, it was observed that transfused subjects were at risk of dying at 30 days showing significant differences (relative risk [RR], 2.3; 95% confidence interval [CI], 1.1–4.9; *P* = .03). Age also showed differences for 30-day mortality (median: 82 vs 79 years, *P* = .02). Likewise, the subjects who underwent HHA and died within 30 days also presented differences (RR, 2.2; 95% CI, 1.1–4.7; *P* = .035). In the same way, in the comparative analysis of the risk of mortality at 30 days in relation to the implant used, it was observed that only the HHA had significant differences in the groups with and without hypothermia and in the rest of the implants there were no differences, ASA risk did not represent a risk for 30-day mortality (Table 3). From the comparative analysis between the risk of hypothermia and the implant used, it was observed that HHA and PFN presented risk for hypothermia and showed statistical differences, the rest of the implants had no differences (Table 4). From the analysis carried out using a Cox regression model of the risk variables for mortality at 30 days after the occurrence of the HF surgery, it was determined that the HHA and the number of globular packages transfused were significant (Table 5). A survival curve with log-rank test was executed to determine differences in the survival of subjects with and without hypothermia (Fig. 1). Likewise, in an analysis between subjects who received from 0 to 3 transfusions there were differences in survival in the 4 groups of the elderly 30 days after having had HF surgery. The log-rank test was χ^2^ = 38.932, *P* < .001.

**Table 1:** Demographic characteristics and relevant comorbidities.

Characteristics	Hypothermia (<35°C) n = 36 (12%)	Euthermia (≥35°C) n = 264 (88%)	RR	95% CI	*P*
Age, yr, median, IQR	82 (75–87.5)	80 (73–84.5)	−	−	.1
Sex (%)					
Male	12 (33.3)	78 (29.5)	1.1	0.7–1.9	.64
Female	24 (66.7)	186 (70.5)			
30-day mortality (%)					
Yes	9 (25)	18 (6.8)	3.7	1.8–7.5	.002
No	27 (75)	246 (93.2)			
SSI (%)					
Yes	4 (11.1)	7 (2.7)	4.2	1.3–13.6	.03
No	32 (88.9)	257 (97.3)			
Fracture type					
Intracapsular	14 (38.9)	94 (35.6)	1.1	0.7–1.7	.7
Extracapsular	22 (61.1)	170 (64.4)			
BMI kg/m^2^, median, IQR	25.3 (21.5–30)	25.1 (23–29.5)			.6
Weight (kg)	68.5 (53.5–78)	63 (58–70)			.7
Diabetes Mellitus	26 (72.2)	165 (62.5)	1.1	0.9–1.1	.2
SAH	27 (75)	164 (62.1)	1.2	0.9–1.5	.1

BMI = body mass index, CI = confidence interval, IQR = interquartile range, RR = relative risk, SAH = systemic arterial hypertension, SSI = surgical site infection.

**Table 2:** Anesthetic risk and surgical characteristics of the elderly population operated for hip fracture exposed to hypothermia.

Characteristics	Hypothermia (<35°C) n = 36 (12%)	Euthermia (≥35°C) n = 264 (88%)	RR	95% CI	*P*
Bleeding ml, median, IQR	200 (100–300)	185 (100–335)	−	−	.5
Transfusion (%)					
Yes	19 (52.8)	28 (10.6)	3.6	2.0–6.5	<.001
No	17 (47.2)	236 (89.4)			
Number of globular packages	1 (1–2)	0 (0–0)			<.001
MAP mm Hg, median, IQR	80 (70–92.5)	80 (75–90)			.9
Time for surgery, days, median, IQR	9 (7.5–10.5)	8 (7–10)	–	−	.1
Operating Time, minutes, median, IQR	60 (47.5–80)	60 (45–85)	−	−	.7
ASA (%)					
I	0 (0)	26 (9.8)	1.1	0.9–1.2	.05
II	9 (25)	143 (54.2)	1.6	1.3–2.1	.001
III	27 (75)	95 (36)	2.1	1.6–2.7	<.001

ASA = American society of anaesthesiologists, CI = confidence interval, IQR = interquartile range, MAP = mean arterial pressure, RR = relative risk.

**Table 3:** Implant used, anesthetic score and risk of mortality at 30 days of hip fracture surgery in elderly exposed and not exposed to hypothermia.

Implants	30-day mortality 27 (9%)	No 30-day mortality 273 (91%)	RR	95% CI	*P*
DHS (%)	11 (40.7)	97 (35.5)	1.2	0.6–2.5	.6
PFN (%)	2 (7.4)	40 (14.7)	0.5	0.1–2.0	.4
HHA (%)	9 (33.3)	46 (16.8)	2.2	1.1–4.7	.03
Plate (%)	1 (3.7)	5 (1.8)	1.9	0.3–11.7	.5
DCS (%)	1 (3.7)	15 (5.5)	0.6	0.1–4.7	.7
THR not cemented (%)	2 (7.4)	37 (13.6)	0.5	0.1–2.2	.4
THR cemented (%)	1 (3.7)	33 (12.1)	0.3	0.05–2.1	.2
ASA (%)					
I	1 (3.7)	25 (9.2)	0.4	0.05–2.9	.3
II	13 (48.1)	139 (50.9)	0.9	0.4–1.9	.8
III	13 (48.1)	109 (39.9)	1.4	0.7–2.8	.4

ASA = American society of anaesthesiologists, CI = confidence interval, DCS = dynamic condylar screw, DHS = dynamic hip screw, HHA = hip hemiarthroplasty, PFN = proximal femoral nail, RR = relative risk, THR = total hip replacement.

**Table 4:** Implant used and risk of hypothermia in the elderly treated for hip fracture.

Implants	Hypothermia (<35°C) n = 36 (12%)	Euthermia (≥35°C) n = 264 (88%)	RR	95% CI	*P*
DHS (%)	12 (33.3)	96 (36.4)	0.9	0.5–1.7	.7
PFN (%)	1 (2.8)	41 (15.5)	0.2	0.02–1.2	.04
HHA (%)	13 (36.1)	42 (15.9)	2.5	1.4–4.6	.003
Plate (%)	1 (2.8)	5 (1.9)	1.4	0.2–8.6	.7
DCS (%)	2 (5.6)	14 (5.3)	1.0	0.3–4.0	.9
THR not cemented (%)	3 (8.3)	37 (13.6)	0.6	0.2–1.9	.6
THR cemented (%)	4 (11.1)	30 (11.4)	0.9	0.4–2.6	.9

CI = confidence interval, DCS = dynamic condylar screw, DHS = dynamic hip screw, HHA = hip hemiarthroplasty, PFN = proximal femoral nail, RR = relative risk, THR = total hip replacement.

**Table 5:** Cox regression to determine the influence of risk factors on 30-day mortality in a cohort of elderly people who had hip fracture surgery with and without exposure to hypothermia.

Step 6	*B*	SE	*χ* ^*2*^	gl	*P*	Exp *B*	95% CI
Hip hemiarthroplasty	0.833	0.424	3.853	1	.049	2.3	1.001–5.285
Number of transfusions	0.973	0.210	21.416	1	<.001	2.6	1.752–3.996

χ^2^ = Ji squared of Wald, B = coefficient, CI = confidence Interval, Exp B = exponent B, gl = liberty degrees, SE = standard error.

## 4 Discussion

As mentioned before, unintentional hypothermia could be a risk factor for mortality in elderly people who undergone HF surgery. As seen in this report, 25% of patients with hypothermia died within 30 days after HF surgery. These results are in discordance, compared to what is reported by Williams et al with 10.9%.^[12]^ This could be due to the fact that in this report there was a larger population of subjects. Also was discordant the incidence of hypothermia with 12%, lower than was reported by Gurunathan et al,^[7]^ of 30%. This, difference could be due to in that report, hypothermia was more frequent in the preoperative period and we only took the temperature in the operation and recovery rooms, which shows that the decrease in temperature is latent, both in pre-anesthesia and in the operation or recovery room, so the use of heating devices should be done several hours before taking the subject to pre-anesthesia and continue to use this heating devices up to the subject final stabilization. In the same way, Wetz et al^[21]^ reported 21.3% of hypothermia in the preoperative period, and age, as a predictive factor, which is consistent with this report, since age was a risk factor for 30-day mortality. In contrast, the incidence of hypothermia in our study was lower (12%), which may be due to the fact that in that report, different sites and methods for measuring temperature were taken into account, as well as, dissimilar surgical procedures, which shows that thermoregulation in the elderly depends on anesthetic factors, the environment, oxygen consumption and heat loss through the skin;^[22]^ and that thermal regulation can be influenced by the health status of the subjects.

A finding that seems relevant to comment, was that the indication for transfusion and the amount of them, were risk factors that had influence over time for 30-day mortality to occur in elderlies who had HF surgery. This is consistent with 2 studies,^[23,24]^ which report that advanced age, sex, ASA risk and long cephalon-medullary nails are predictive factors for intraoperative transfusion, and that 30-day mortality is higher in the elderly who required transfusion 5% vs 3.7% (RR, 12.3; 95% CI, 11.9–13.1). Was not observe a higher frequency of transfusion with any of the implants used (*P *> .05), but we did observe, in an unprecedented way, that subjects undergoing surgical treatment with PFN were at risk of hypothermia, because with this type of implant and/or surgical technique in addition to axial or general anesthesia, heat is likely to be lost in the lower extremities when uncovered during surgery. This can be explained by the redistribution of central heat due to peripheral vasodilation in the lower extremities which causes linear reduction of the central temperature to keep the extremities warm, resulting in intraoperative heat loss through radiation and convection that exceeds the metabolism's heat production.^[25]^ This could lead to more reports in the future.

On the other hand, the second most used implant in this report was HHA with a 18.3%. It was observed that the subjects undergoing HHA had a higher risk of mortality 30 days after the occurrence of HF and that they were also older subjects (*P *< .001). Likewise, it found that the subjects classified with ASA III underwent HHA more frequently because it is likely that patients with a worse state of health, low ambulatory ability and old age, could die in the short term; for this reason, in current practice, indication for HHA is limited to this type of subjects.^[26]^ However, in a recent study conducted in ambulatory subjects at 24 months of evolution with HHA vs THR, it has been reported that there are no statistical differences in mortality, in adverse events or in stability and that there are few advantages in favour of THR in terms of pain, functionality and stiffness.^[27]^ This shows that HHA should not be reserved only for subjects with poor health or who are older, and that the ideal surgical management remains uncertain and should continue in debate because extending the indication for HHA in younger subjects with better health, could help to reduce costs in public health systems.

On the other hand, hypothermia in this study was also a risk factor for SSI. The proposed mechanism is that hypothermia affects lymphocyte circulation and the expression of anti-inflammatory molecules.^[28]^ However, in a recent meta-analysis, it is suggested that intraoperative hypothermia is not associated with an increased risk of SSI in non-orthopaedic surgery.^[29]^ However, in a recent report in subjects undergoing surgery for calcaneal fractures, hypothermia was 1.7 times higher risk factor for SSI.^[30]^ Evenly, in spinal fusion surgery, inadvertent hypothermia was frequent, so it is recommended to maintain euthermia to avoid complications such as SSI, bleeding, and cardiac morbidity.^[31]^ Further studies are required to prove that major orthopaedic surgery predisposes to inadvertent hypothermia.

Other findings to highlight, was that although severely ill subjects were not included, patients, classified as ASA II and III, had 1 to 2 times higher risk of hypothermia, but without being a factor for 30-day mortality in HF, since was not found differences in the 30-day survival in groups ASA I, II, and III. But a report refers,^[21]^ in subjects ASA IV have 6 times the risk of hypothermia, but without being an independent factor associated with hypothermia. Equally, another recent study^[32]^ observed a weak linear association between ASA and hypothermia and also refers that ASA is not a predictor for hypothermia. In opposition, Li et al,^[33]^ argue that the mortality at 1 year after the occurrence of HF is higher in ASA III, IV, and V patients, which reflects that a severe comorbid state in the elderly is usually accompanied by HF, which is a geriatric syndrome and a predictor of mortality.

The weakness in this report is where the temperature was taken, since the rectal temperature has not yet been validated for the diagnosis of hypothermia, and because in the elderly it is common to have rectal hypoperfusion, which possibly had an influence on producing lower temperatures with the resultant lower temperature readings relative to other sites, causing sensitivity bias. In addition, the preoperative temperature and the time between the initial intraoperative measurement and the arriving to the recovery room were not taken; and as a result, temperature fluctuations were not compared, which could cause a measurement bias with incorrect non-differential classification. Likewise, although there were no differences in intraoperative time for HF fixation, the variability between the implants used and the skill of the surgeons are factors that should be taken into consideration for mortality. The strengths of this research focus on the fact that it was a prospective study, that the temperature measurement was carried out by blind research personnel and that ASA IV and V subjects or patients with severe comorbid status were not included to avoid increased incidence in 30-day mortality.

## 5 Conclusion

Elderly subjects with HF, exposed to hypothermia, who undergo HHA, and who receive 2 or more blood transfusions during their treatment, are at greater risk of dying within 30 days of having surgery. We recommend continuous temperature monitoring to be able to implement actions to prevent hypothermia, and in this way, to avoid other risk factors that influence short-term mortality. Hypothermia as a possible causative factor of mortality should continue to be studied because its origin and influence on mortality in the elderly with HF can be multifactorial.

## Author contributions

**Conceptualization:** Sergio Charles-Lozoya.

**Data curation:** Jesús Mario Rangel-Valenzuela.

**Formal analysis:** Sergio Charles-Lozoya, Miguel Leonardo De La Parra-Márquez.

**Investigation:** Sergio Charles-Lozoya.

**Methodology:** Sergio Charles-Lozoya, Héctor Cobos-Aguilar, Edgar Manilla-Muñoz, Adrián García-Hernández.

**Resources:** Adrián García-Hernández.

**Supervision:** Miguel Leonardo De La Parra-Márquez, Adrián García-Hernández.

**Writing – original draft:** Sergio Charles-Lozoya.

**Writing – review & editing:** Sergio Charles-Lozoya, Héctor Cobos-Aguilar, Edgar Manilla-Muñoz, Jesús Mario Rangel-Valenzuela.
